# Enhanced Placental Mitochondrial Respiration in Tibetan Women at High Altitude

**DOI:** 10.3389/fphys.2021.697022

**Published:** 2021-07-14

**Authors:** Huifang Liu, Noryung Tenzing, Martha Tissot van Patot, Muge Qile, Ri-li Ge, Tana Wuren

**Affiliations:** ^1^Research Center for High Altitude Medicine, Qinghai University, Xining, China; ^2^Key Laboratory for Application of High-Altitude Medicine, Qinghai University, Xining, China; ^3^Affiliated Hospital of Qinghai University, Xining, China; ^4^Retired, Fort Collins, CO, United States

**Keywords:** Tibetan, mitochondria respiration, placenta, hypoxia, high altitude adaptation

## Abstract

Living at high altitudes is extremely challenging as it entails exposure to hypoxia, low temperatures, and high levels of UV radiation. However, the Tibetan population has adapted to such conditions on both a physiological and genetic level over 30,000–40,000 years. It has long been speculated that fetal growth restriction is caused by abnormal placental development. We previously demonstrated that placentas from high-altitude Tibetans were protected from oxidative stress induced by labor compared to those of European descent. However, little is known about how placental mitochondria change during high-altitude adaptation. In this study, we aimed to uncover the mechanism of such adaptation by studying the respiratory function of the placental mitochondria of high-altitude Tibetans, lower-altitude Tibetans, and lower-altitude Chinese Han. We discovered that mitochondrial respiration was greater in high-altitude than in lower-altitude Tibetans in terms of OXPHOS via complexes I and I+II, ETS_max_ capacity, and non-phosphorylating respiration, whereas non-ETS respiration, LEAK/ETS, and OXPHOS via complex IV did not differ. Respiration in lower-altitude Tibetans and Han was similar for all tested respiratory states. Placentas from high-altitude Tibetan women were protected from acute ischemic/hypoxic insult induced by labor, and increased mitochondrial respiration may represent an acute response that induces mitochondrial adaptations.

## Introduction

The oxygen content of the atmosphere is 21% regardless of altitude, while the barometric pressure decreases as the altitude increases. In medical research, high altitude is defined as ≥2,500 m above sea level (Julian and Moore, 2019). The main challenges at high altitudes are hypoxia, low temperatures, and high levels of UV radiation (Peacock, 1998; Butaric and Klocke, 2018; Song et al., 2020). The Tibetan population is considered both physiologically and genetically adapted to such hypoxic conditions since they have lived and successfully reproduced at high altitudes for 30,000–40,000 years (Zhang et al., 2018; Bhandari and Cavalleri, 2019). Tibetans are protected from polycythemia, pulmonary hypertension, low birth weight, and other hypoxia-related diseases that normally result from prolonged exposure to such inhospitable environments (Yang et al., 2017; Jeong et al., 2018; Bhandari and Cavalleri, 2019; Song et al., 2020). Most research has focused on Tibetans living permanently at high altitudes, and there have been few studies of Tibetans living at relatively low altitudes. Therefore, we decided to devote a study to the differences between lower- and high-altitude Tibetans, as well as between lower-altitude Tibetans and other ethnic groups, such as Chinese Han, living at the same altitude.

At high altitudes, an insufficient maternal supply of O_2_ and nutrients leads to fetal growth restriction (Bailey et al., 2019; Lorca et al., 2019, 2020; Lane et al., 2020a,b). Fetal growth restriction is a condition in which the fetus fails to reach its full growth potential, related to the increased risk of development of metabolic syndrome in childhood and multiple diseases in adulthood; therefore, it has received increasing attention (de Rooij et al., 2007; Veenendaal et al., 2013; Sayama et al., 2020). The placenta plays an essential role in fetal growth (Shaw et al., 2018; Sferruzzi-Perri et al., 2019). It has long been speculated that fetal growth restriction is initially caused by abnormal placental development and functional deficiency (Tong and Giussani, 2019; Sangkhae et al., 2020; Turner et al., 2020). Most Tibetans experience unrestricted fetal growth at high altitudes and our previous study indicated that placentas of Tibetans living at high altitudes are protected from labor-induced oxidative stress compared to those of other high-altitude residents (Tana et al., 2021). Placental mitochondria are critical to the healthy development of the fetus; however, little is known about mitochondrial respiration in populations adapted to high altitudes. Impaired placental mitochondrial function can have a detrimental effect on fetal development (Tissot van Patot et al., 2010). Therefore, etiologic studies of hypoxia-related pregnancy diseases, such as pre-eclampsia and intrauterine growth restriction, should include focus on the mitochondria.

The aim of this study was to investigate mitochondrial respiration in Tibetan women permanently residing at high altitudes and compare it with those permanently residing at lower altitudes. We hypothesized that Tibetan placenta possesses enhanced mitochondria respiration capacity to remedy the increased energy demand of labor and oxidative stress during the labor in the oxygen deprived environment. This was done in the attempt to further our understanding of the mechanism of adaptation to high altitudes and the risk factors that change the growth trajectory of the fetus. Ultimately, we strived to identify opportunities to improve the health of children and reduce the risk of many adult diseases related to fetal growth restriction.

## Materials and Methods

### Materials and Sample Collection

All chemical substrates were purchased from Sigma Aldrich, Australia. This study was approved by the Qinghai University Affiliated Hospital Ethics Committee. In total, 21 human full-term (37–40 weeks) placentas were collected after laboring vaginal delivery, with the written consent of donors after receiving an explanation from local doctors in their native language. Placental tissues from high-altitude Tibetan women (*n* = 9) were collected at Yushu Bayi Hospital (3780 m); those from lower-altitude Tibetan (*n* = 7) and Han (*n* = 5) woman were collected at the Guide County Hospital (2200 m) and Qinghai University Affiliated Hospital (2261 m). The general characteristics of the pregnancies are provided in Table 1. All neonates had Apgar scores between 7 and 9.

**Table 1:** Maternal and infant characteristics.

	**Tibetan**		**Chinese Han**	***P*****-values**	
	**3780 m**	**2200 m**	**2200 m**	**Altitude**	**Nationality**
**Maternal characteristics**					
n	9	7	5		
Age (years)	28.2 ± 2.4	26.6 ± 1.8	28.6 ± 2.1	NS	NS
Parity	3.3 ± 0.7	1.7 ± 0.3	1.8 ± 0.4	NS	NS
Height (cm)	161 ± 2	167 ± 1	165 ± 2	<0.05	NS
Non-pregnant weight (kg)	55 ± 3	54 ± 2	61 ± 4	NS	NS
Non-pregnant body mass index (kg m^−2^)	34 ± 2	33 ± 1	37 ± 3	NS	NS
Weight gain with pregnancy (kg)	13 ± 1	13 ± 2	14 ± 2	NS	NS
Systolic BP (mmHg)	111 ± 2	108 ± 2	103 ± 5	NS	NS
Diastolic BP (mmHg)	72 ± 2	66 ± 2	66 ± 3	NS	NS
**Infant characteristics**					
Birth weight (g)	3318 ± 125	3253 ± 150	3423 ± 186	NS	NS
Birth/Placental weight ratio	5.5 ± 0.2	5.6 ± 0.3	5.7 ± 0.4	NS	NS
Apgar score	8.778 ± 0.443	8.857 ± 0.35	8.8 ± 0.4	NS	NS

*All values are either numbers or means ± standard errors of the mean. BP, blood pressure*.

Each placenta was weighed immediately after delivery and divided into six sections for random sampling. Samples from the fetal side of the placenta were biopsied within 15 min, placed on ice and in PBS, and delivered to the laboratory.

### High-Resolution Respirometry

In order to most accurately assess the metabolic characteristics of mitochondrial activity in placentas delivered at high altitude, we installed a respirometer in a laboratory at the delivery site. The respirometer was carefully recalibrated for optimal accuracy in the low-O_2_ environment.

The samples were washed in ice-cold PBS to remove blood. The plasma membranes were permeabilized for 40 min on ice and mixed with 50 μg/mL saponin in 1 mL biopsy preservation solution containing CaK_2_EGTA (2.77 mM), K_2_EGTA (7.23 mM), Na_2_ATP (5.77 mM), MgCl_2_·6H_2_O (6.56 mM), taurine (20 mM), sodium phosphocreatine (15 mM), imidazole (20 mM), dithiothreitol (0.5 mM), and MES (50 mM) at pH 7.1. The samples were washed twice for 10 min on ice in mitochondria respiration medium (MiR05) containing 0.5 mM EGTA, 3 mM MgCl_2_·6H_2_O, 60 mM K-lactobionate, 20 mM taurine, 10 mM KH_2_PO_4_, 20 mM HEPES, 110 mM sucrose, and 1 g/L BSA (fatty acid free) at pH 7.1, after which the tissue was used for respirometry.

**Figure 1:**
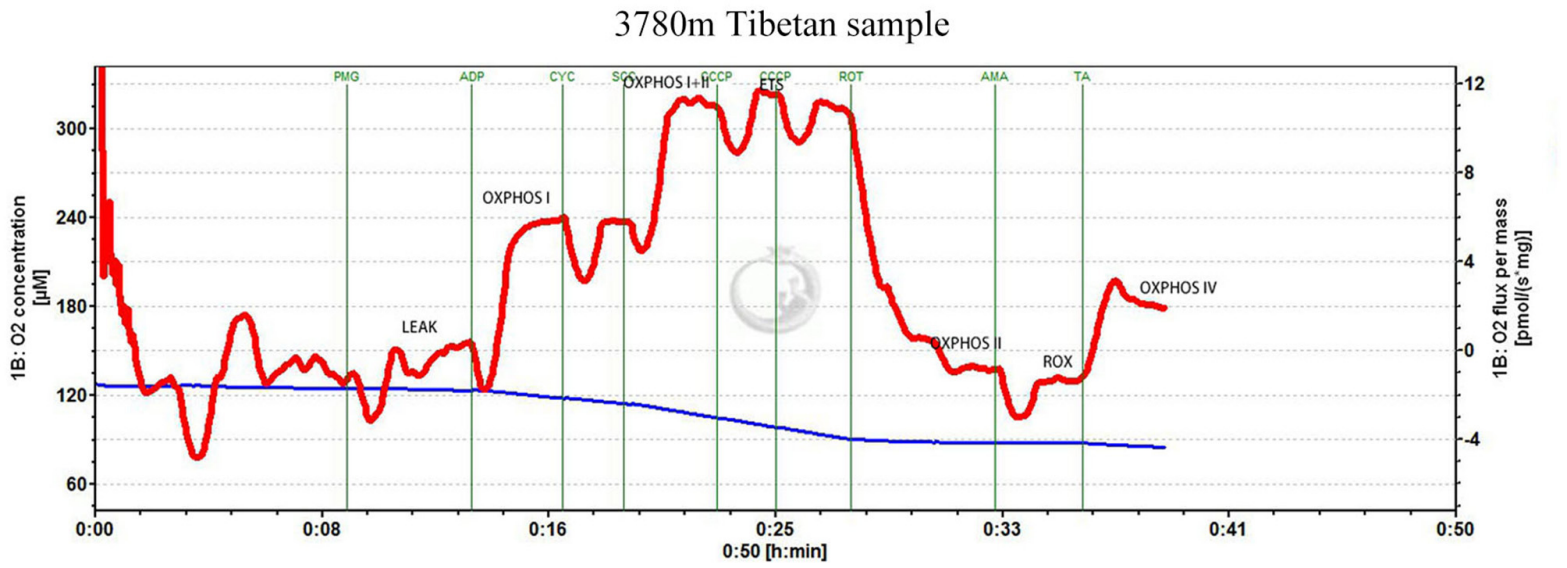
Substrate-uncoupler-inhibitor titration (SUIT) protocol. Representative oxygraph traces of oxygen flux relative to tissue mass. The vertical lines show the introduction of various substrates or inhibitors. LEAK refers to non-phosphorylating respiration; OXPHOS CI, CII, and CIV represent oxidative phosphorylation through mitochondrial complexes I, II, and IV, respectively; ETS_max_ represents electron transfer system maximum capacity; ROX represents non-ETS respiration; PMG represents pyruvate, glutamate, and malate; CYC represents cytochrome c; ROT represents rotenone; SCC represents succinate; CCCP represents the uncoupler, 2-[(3-chlorophenyl)hydrazinylidene]propanedinitrile (CCCP); AMA represents antimycin A; TA represents 1-*N*,1-*N*,2-*N*,2-*N*-tetramethylbenzene-1,2-diamine;dihydrochloride and ascorbate.

To measure mitochondrial respiration, 5–10 mg wet-weight tissue was added to the chamber of an Oxygraph-2k respirometer (Oroboros Instruments, Austria) containing MiR05 at 37°C, and a substrate-uncoupler-inhibitor titration protocol was used, as illustrated in Figure 1 (from a high-altitude Tibetan sample) (Supplementary Figure 1A for low altitude Tibetan and Supplementary Figure 1B for low altitude Han).

Glutamate (10 mM), malate (5 mM), and pyruvate (5 mM) were added to stimulate non-phosphorylating (LEAK) respiration mediated by complex I (CI). Subsequently, ADP (5 mM) was added to activate the phosphorylation of ADP to ATP through CI. Cytochrome c (10 μM) was added to confirm the integrity of the outer mitochondrial membrane; Succinate (10 mM) was added to stimulate oxidative phosphorylation (OXPHOS) through complex II (CII). Carbonyl cyanide m-chloro phenyl hydrazine (stepwise titration of 1 mM) was used to uncouple OXPHOS and investigate the capacity of the electron transfer system (ETS). CI was inhibited using rotenone (1 mM), to separately determine the succinate-linked ETS capacity. Complex III was inhibited using antimycin A (2.5 μM) to determine the residual O_2_ consumption. Finally, 1-*N*,1-*N*,2-*N*,2-*N*-tetramethylbenzene-1,2-diamine; dihydrochloride (0.5 mM) and ascorbate (2 mM) were added to measure OXPHOS through complex IV (CIV).

Thus, the main outcome measures were LEAK respiration and OXPHOS capacity through CI, CII, ETS maximum capacity, and CIV. The respiratory control ratio (RCR) was calculated as OXPHOS capacity with CI substrates and saturating ADP as a fraction of LEAK respiration with CI substrates.

### Statistical Analysis

GraphPad Prism version 8 (GraphPad Software, San Diego, California, USA)^1^ was used for statistical analyses. Values are expressed as means ± standard errors of the mean. Data were analyzed using the D'Agostino-Pearson omnibus normality test, and Student's *t*-tests were used to evaluate the differences between two groups at a time. Statistical significance was defined as *p* < 0.05.

## Results

### Respiration of Placental Mitochondria at Different Altitudes

**Figure 2:**
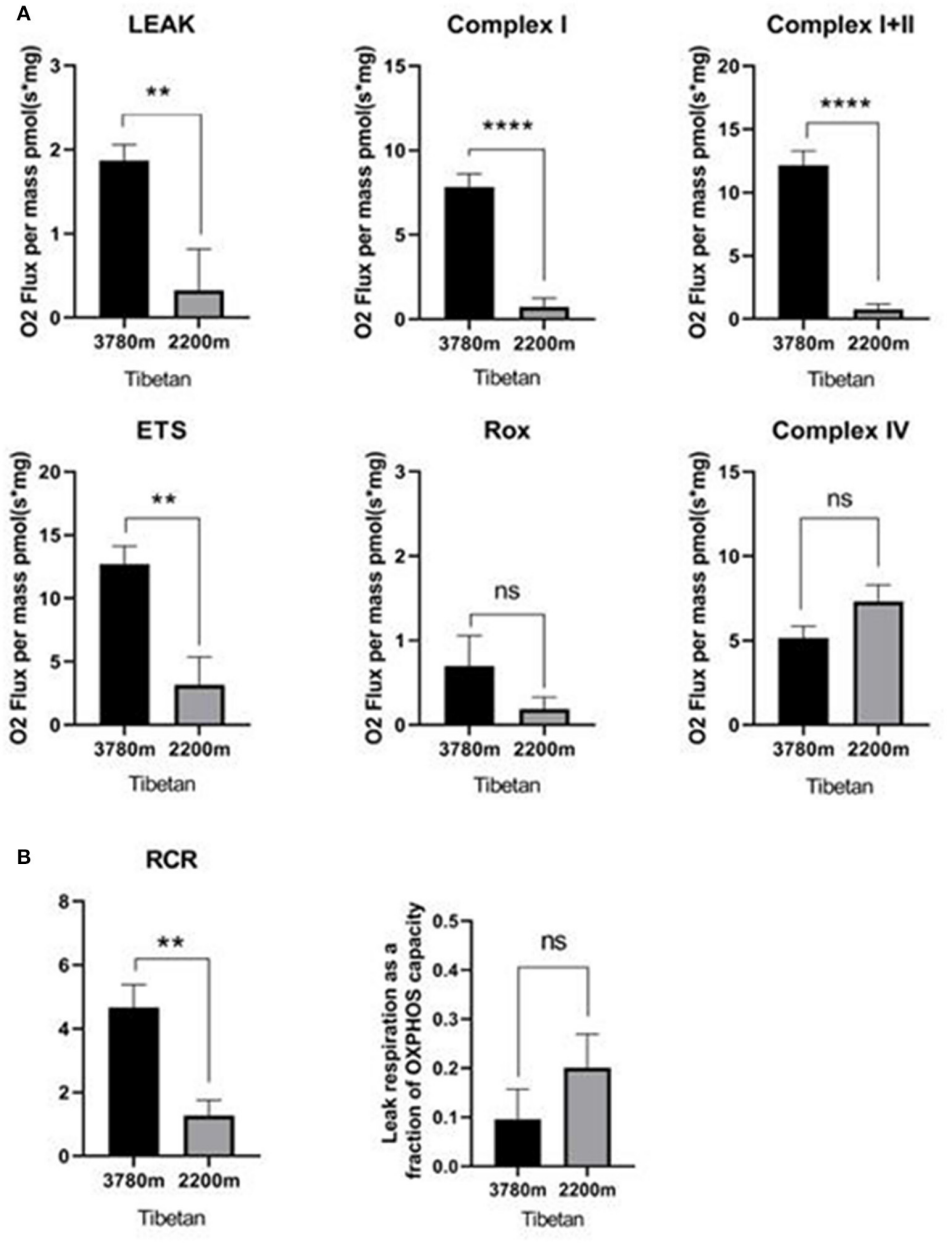
Respiration in high- vs. lower-altitude Tibetan groups. **(A)** Oxidative phosphorylation (OXPHOS) through mitochondrial complex I, complex I+II, non-phosphorylating (LEAK) respiration, and electron transfer system (ETS) maximum capacity respiration were statistically significantly increased in high-altitude Tibetan group compared to lower-altitude Tibetan group. Non-ETS respiration (ROX) and OXPHOS through mitochondrial complex IV did not change with altitude. **(B)** The respiratory control ration (RCR) was statistically significantly increased in the high-altitude Tibetan group compared to the lower-altitude Tibetan group. But the LEAK/ETS was not significant change. ns: *p* ≥ 0.05; ^**^p < 0.01; ^****^p < 0.0001. Statistical significance was determined via Student's *t*-test. Columns represent means and error bars present standard errors of the mean.

As illustrated in Figure 2A, the mitochondrial respiration capacity in the high-altitude Tibetan group was greater than that in the lower-altitude Tibetan group in terms of OXPHOS with substrates for CI and CI+II, as well as for ETS maximum capacity and LEAK. Non-ETS respiration and OXPHOS with substrates for CIV, however, remained stable across different altitude Tibetan groups. As displayed in Figure 2B, the RCR in the high-altitude Tibetan group was higher than that in the lower-altitude Tibetan group, LEAK/ETS was not change, which suggests stronger coupling of mitochondrial respiration and ATP synthesis in the high-altitude Tibetan group.

### Respiration of Placental Mitochondria in Different Nationality

**Figure 3:**
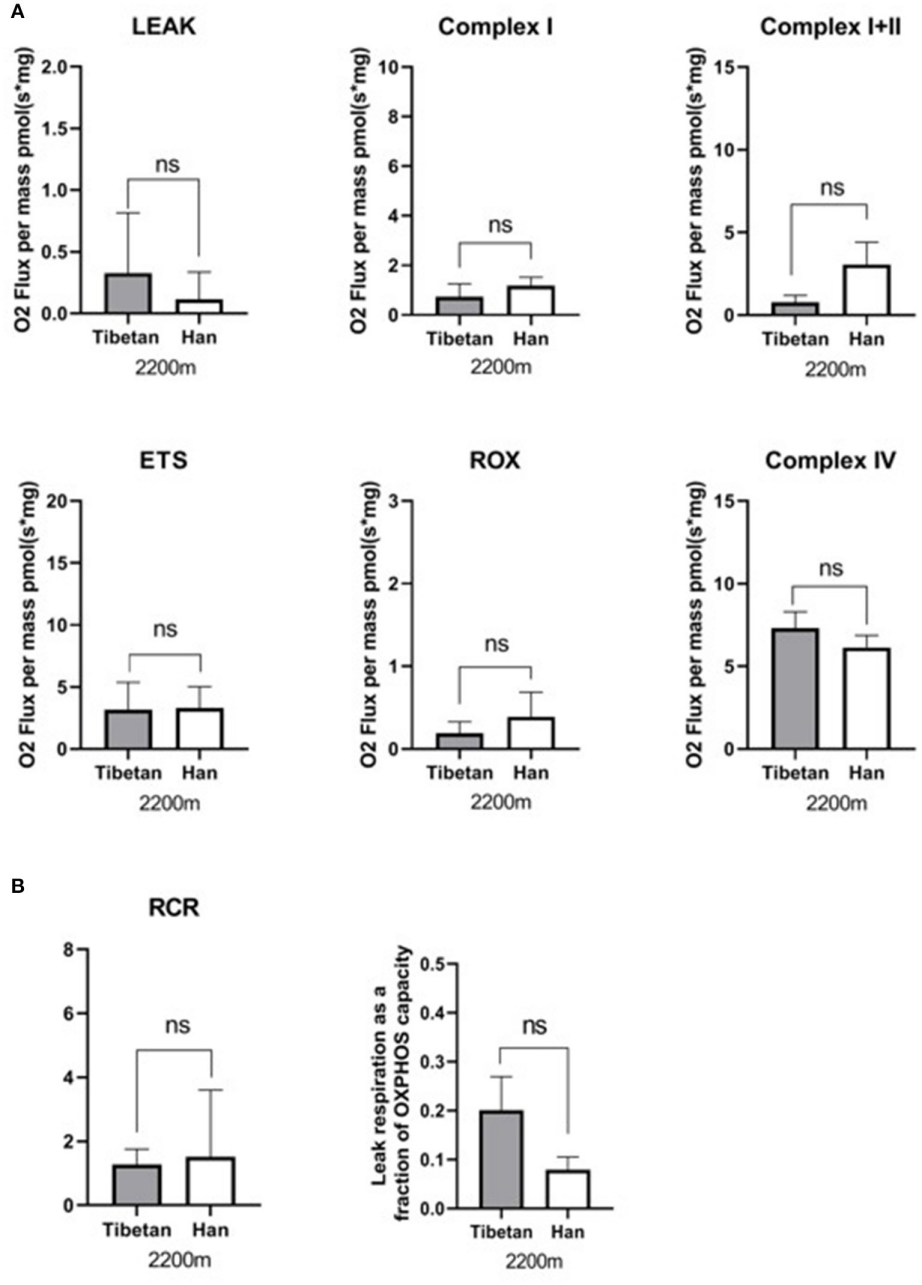
Respiration in lower-altitude Tibetan vs. Han groups. **(A)** Oxidative phosphorylation (OXPHOS) with substrates for mitochondrial complexes I (CI), C I+II, and CIV; non-phosphorylating respiration (LEAK); ETS maximum capacity respiration; and non-ETS respiration (ROX) were not statistically significantly different between the lower-altitude Tibetan and Han groups. ns: *p* ≥ 0.05. **(B)** The respiratory control ration (RCR) and LEAK/ETS was not statistically significantly different between the lower-altitude Tibetan and Han groups.

Respiration in the lower-altitude Tibetan and Han groups was similar for all measured respiratory states (Figure 3). The respiratory function of mitochondria in Tibetan and Han women at the same altitude and mode of delivery was similar. We observed a low RCR in lower-altitude Tibetan (RCR: 1.4) and Han (RCR: 1.5) mitochondria using substrates for CI, suggesting that these mitochondria have impaired respiratory efficiency.

## Discussion

Our study provides evidence that significant differences in human placental mitochondrial respiration exist between Tibetan women living at high and those at lower altitudes. Specifically, through the utilization of mitochondrial complexes.

The energy metabolism of cardiac and skeletal muscles is altered at high altitudes. Hypoxic rats (13% O_2_, 2 weeks) exhibited a loss of fatty acid oxidation capacity, complex I-supported respiration, and ATP levels in the left ventricle, and experienced increased oxidative stress (Ashmore et al., 2014). Further investigations have been carried out on the metabolic response of human skeletal muscle at high altitudes. At moderately high altitudes, respiratory capacity of fatty acid oxidation decreased without changes in mitochondrial volume density (Murray, 2016). In high altitude residents, pregnant women had increased O_2_ demand and blood O_2_ saturation compared to pregnant women at sea level. However, the decrease in arterial blood oxygen content during high-altitude pregnancies may lead to physiologic anemia due to the expansion of circulating plasma volume in the second trimester, which is also related to an insufficient increase in cardiac output. Interestingly, it was observed that women who lived on the Tibetan plateau for less than three generations had less hemoglobin and arterial blood O_2_ content during pregnancy, compared with those who live in a multi generations there (McAuliffe et al., 2001). Tibetan women at high altitudes also have a greater uterine artery blood flow than women at low altitude, providing more O_2_ and nutrients to the fetus (Moore, 1990). There is evidence to suggest that they may also have an increased mitochondrial activity and a larger RCR to use O_2_ more efficiently, ensuring sufficient growth of the fetus and a higher birth weight (Moore, 1990).

Our results suggest that mitochondrial respiration was higher in all tested states, except for non-ETS and CIV-mediated respiration, in the high- compared to the lower-altitude Tibetan group. The RCR was also greater in the high- as compared with the lower-altitude Tibetan group, suggesting improved coupling to ATP production and/or less oxidative damage. A decrease in the RCR often coincides with elevated oxidative stress. These findings are consistent with those of Bustamante et al. (2014). The respiratory function of mitochondria at lower altitudes was less than that in high-altitude Tibetans. This may be related to the oxidative stress experienced by mitochondria in response to labor-induced placental ischemia and reperfusion. However, previous studies have indicated a decrease in placental ATP (Bloxam and Bobinski, 1984) and an increase in placental oxidative stress markers during vaginal delivery (Diamant et al., 1980; Cindrova-Davies et al., 2007). The placental mitochondria of high-altitude Tibetans appear to make more efficient use of O_2_ than do those of lower-altitude Tibetans when there is oxygen shortage at higher altitude, which may be the outcome of adaptation to high altitudes. Mitochondria in the skeletal muscles have been demonstrated to adapt to strength and endurance training (Zoll et al., 2002; Pesta et al., 2011). Changes in placental mitochondria have been reported for several disease states (Mandò et al., 2014; Mele et al., 2014; Holland et al., 2017), many of which are thought to include hypoxia/reperfusion injury (Holland et al., 2017). Our data indicate that mitochondrial respiration is less robust in Tibetans residing at an altitude of 2,200 m as compared with those living at 3780 m. Sufficient O_2_ supply, a high partial pressure of arterial O_2_, and sufficient uterine artery blood flow maintain the physiological needs of the fetus without increasing the activity of mitochondria. Additionally, our previous study suggested that placentas from Tibetan women living at high altitudes were better protected from labor-induced oxidative stress than were high-altitude residents of European descent (Tana et al., 2021). The reproductive success of Tibetans is therefore likely to be, at least in part, due to cardiac-related traits and placental adaptation, via increased mitochondrial respiration (Burton et al., 2016; Jeong et al., 2018).

Our data suggests that the result might be of genetic selection. We intend to examine the correlation between gene variants, fetal birth weight and placental function. Limited by the interrelatedness of the geographical location, environment surroundings and one's ethnic cultural background, very few Han women are known to live at high altitude during their pregnancy so that collecting appropriate placental specimens and data of the high plateau Han women became extremely difficult for us. Meanwhile our study is also restricted by the fact that as majority of Tibetan women are permanent residents at an altitude above 2000 m, the chance of them living at sea level was rare, therefore recruiting sea level Tibetan pregnant women for our research had been impossible for years. That being said, our study still provides strong evidence that the adaptation of mitochondrial respiration in high altitude Tibetans is likely to contribute to reproductive success at plateau.

## Data Availability Statement

The original contributions presented in the study are included in the article/Supplementary Material, further inquiries can be directed to the corresponding author.

## Ethics Statement

The studies involving human participants were reviewed and approved by Qinghai University Affiliated Hospital Ethics Committee. The patients/participants provided their written informed consent to participate in this study.

## Author Contributions

TW designed the experiments. HL, NT, and MQ performed the experiments and HL and TW analyzed the data. HL, NT, MP, TW, and R-lG wrote, read, edited, and approved the final version of manuscript. All authors contributed to the article and approved the submitted version.

## Conflict of Interest

The authors declare that the research was conducted in the absence of any commercial or financial relationships that could be construed as a potential conflict of interest.

## Footnotes

^1^www.graphpad.com.
